# 7S RNA is surveilling mitochondrial DNA transcription

**DOI:** 10.1093/lifemeta/loac008

**Published:** 2022-06-16

**Authors:** Hao Wu, Ling-Ling Chen

**Affiliations:** State Key Laboratory of Molecular Biology, Shanghai Key Laboratory of Molecular Andrology, CAS Center for Excellence in Molecular Cell Science, Shanghai Institute of Biochemistry and Cell Biology, University of Chinese Academy of Sciences, Chinese Academy of Sciences, Shanghai, China; State Key Laboratory of Molecular Biology, Shanghai Key Laboratory of Molecular Andrology, CAS Center for Excellence in Molecular Cell Science, Shanghai Institute of Biochemistry and Cell Biology, University of Chinese Academy of Sciences, Chinese Academy of Sciences, Shanghai, China; School of Life Science and Technology, ShanghaiTech University, Shanghai, China; School of Life Science, Hangzhou Institute for Advanced Study, University of Chinese Academy of Sciences, Hangzhou, China

**In the recent article published in *Cell*, Zhu and colleagues identified the noncoding RNA, 7S RNA, that couples the homodimer formation of mitochondrial RNA polymerase POLAMT, preventing the latter from interacting with promoter and transcription complex on mitochondrial DNA, leading to suppressed transcription initiation. This negative transcription regulation is modulated by the mitochondrial exoribonuclease complex that can degrade 7S RNA.**

RNA transcription is the critical biological process by which the genomic DNA is copied into RNA sequence. In eukaryotes, the RNA polymerase II (Pol II) transcribes protein-coding and noncoding genes with the help of transcription factors, including general components like TFIIA, TFIIB, TFIID, TFIIE, TFIIF, and TFIIH [1]. The Pol II transcribed coding RNAs are m^7^G capped and polyadenylated, subsequently exported into cytosol, and translated into proteins. Transcription regulation of specific genes is remarkably balanced by enhancer or silencer sequences *in cis* embedded in DNA sequences, and *in trans* by auxiliary factors such as promoter-specific activators, suppressors, and co-regulators [2]. For a long time, these regulatory factors are thought to be proteins and protein-containing complexes, but now it is getting increasingly clear that noncoding RNAs (ncRNAs) also play crucial roles in these processes [3, 4].

Previous studies indicate that eukaryotic cells upon stringent stimulations, such as heat shock or virus infection, can repress Pol II transcription via upregulation of ncRNAs transcribed by RNA polymerase III (Pol III), mainly B2 and *Alu* RNAs transcribed from short interspersed elements (*SINEs*) [1, 5]. These short ncRNAs interact with the Pol II core domain, change its structural conformation, and prevent Pol II from contacting the upstream and downstream of the protomer TATA box [1]. In addition, prokaryotic 6S RNA can block the active site of prokaryotic RNA polymerase holoenzyme by forming a scaffold structure that directly interacts with the enzyme, subsequently causes repression of σ^70^-promoter dependent RNA expression [6]. However, the ncRNA regulator(s), if any, of mitochondrial DNA (mtDNA) transcription has remained far from clear. In the recent *Cell* article, Zhu *et al*. reported that mitochondrial 7S RNA plays an essential role in controlling mammalian mtDNA transcription [7].

**Figure 1:**
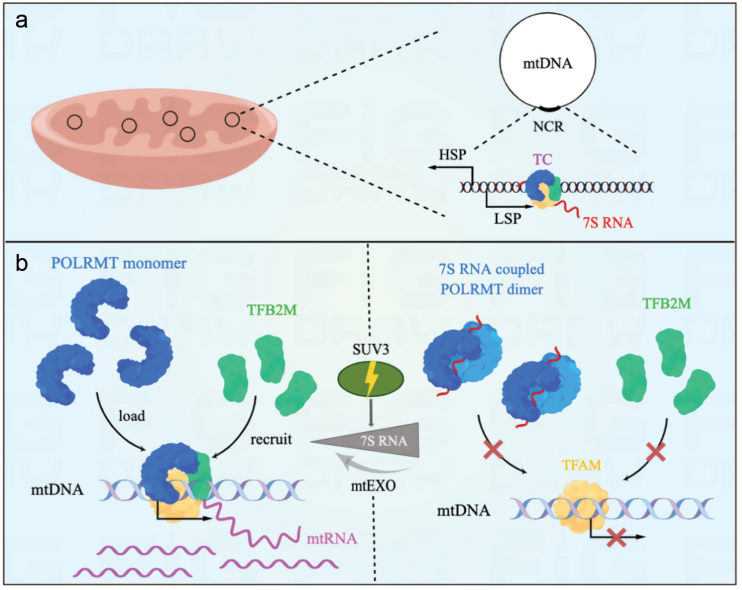
A schematic model of how 7S RNA functions in mitochondrial DNA transcription initiation [7]. (a) Mitochondrial DNA (mtDNA) produces a ~200 nt noncoding RNA, 7S RNA, from the L-strand promotor (LSP) of the non-coding region (NCR). TC, transcription complex. (b) 7S RNA suppresses mtDNA transcription initiation. Left, the monomeric mitochondrial RNA polymerase POLRMT is recruited on the mtDNA promotor by mitochondrial transcription factor A (TFAM). This allows to recruit the second transcription cofactor (TFB2M) to form mitochondrial transcription complex and initiate transcription of mitochondrial RNA (mtRNA). Right, binding 7S RNA enables to form POLRMT homodimer, which is barely loaded on the transcription site, leading to suppressed mtDNA transcription initiation. Of note, this 7S RNA-induced mtDNA transcription regulation is modulated by the mitochondrial exoribonuclease complex (mtEXO). Loss of the mtEXO component, SUV3, results in 7S RNA accumulation and suppressed transcription.

The single unit of mitochondrial genome transcribes into a polycistronic RNA that encodes 13 components of the oxidative phosphorylation system as well as rRNAs and tRNAs required to translate mitochondrial genes [8]. Compared with eukaryotic Pol II transcription machinery, mtDNA transcription is relatively simple. It is initiated by binding the mitochondrial transcription factor A (TFAM) to the upstream sequence of the transcription start site of mtDNA. The TFAM-DNA complex recruits the mitochondrial RNA polymerase POLRMT to the promotor region, which on one hand recruits the second transcription cofactor (TFB2M) to help DNA melting, and on the other hand initiates DNA transcription [9]. Zhu *et al*. reported that the evolutionally conserved, stoichiometrically abundant, mitochondrial 7S RNA is derived from the L-strand promotor (LSP) on mtDNA (Fig. 1a) [7]. 7S RNA bridges POLRMT homodimerization, such that preventing the latter from contacting with promoter, thereby inhibiting mtDNA transcription initiation (Fig. 1b) [7]. The authors have carried out a number of well-designed biochemical and cellular assays to support this key conclusion [7]. In brief, by performing *in vitro* transcription and pulse-chase assays, they found that 7S RNA served as a component of a negative feedback loop modulating mtDNA transcription initiation, but not transcription elongation. The direct interaction between 7S RNA and POLRMT was confirmed by gel filtration and RNase I footprinting assays. Intriguingly, 7S RNA fluorescence *in situ* hybridization (FISH) in cells revealed that loss of SUV3, a component of mitochondrial exoribonuclease complex (mtEXO), led to 7S RNA accumulation and reduced mtDNA transcription. Moreover, with single particle cryogenic electron microscopy (Cryo-EM), they solved the structure of 7S RNA-POLRMT complex at 3.3 Å, showing POLRMT dimer with 7S RNA, which bound POLRMT via nine positively charged residues on the enzyme. Lastly, DNase I footprinting experiments showed that such dimerization blocked POLRMT binding to TFB2M, TFAM, as well as LSP transcription start site (TSS) and −60 to −65 region on mtDNA. Together, results from these extensive technologies have convincingly shown that 7S RNA is an essential negative regulator in mtDNA transcription initiation. Unlike modes of ncRNAs modulating eukaryotic Pol II or prokaryotic RNA polymerase transcription by touching the catalytical active sites or altering the structural conformation of the enzyme complexes [1, 6], 7S RNA suppresses mitochondrial RNA transcription initiation by trigging POLRMT dimerization. Interestingly, transcription elongation by POLRMT is unlikely inhibited by 7S RNA [7]. This is presumably because once the POLRMT transcription complex is assembled, 7S RNA binding sites on the mono-POLRMT are blocked.

It is generally acknowledged that human mitochondrial genome encodes 13 proteins, 2 rRNAs, and 22 tRNAs [8]. However, the transcriptomic complexity within the mitochondria has remained underestimated. In the last decade, long ncRNAs, double-stranded RNAs, small ncRNAs, and circular RNAs have been identified in mitochondria [8], and some have been shown as critical regulators in mitochondrial homeostasis and metabolism [10, 11]. In this paper, Zhu *et al*. have clarified that 7S RNA modulates mtDNA transcription initiation [7], providing strong evidence for yet another regulatory ncRNA in mitochondrial biology. Interestingly, the authors have also shown that 7S RNA can be degraded by the mtEXO [7], whose activity is tightly associated with the length of RNA poly(A) tails [12]. Since the poly(A) length of RNA depends on the cellular Pi/ATP ratio, physiopathological changes affecting oxidation phosphorylation can alter the catalytical activity of mtEXO, thereby likely altering 7S RNA abundance. The working model described in this article [7], to some extent, explains the negative regulation of mitochondrial transcriptome under pathological processes. How mtEXO regulates 7S RNA turnover, and whether mtEXO affects other mitochondrial RNAs that contribute to transcription regulation warrant further studies.

Nonetheless, Zhu *et al*., for the first time, put the 7S RNA in the center in the surveillance of mitochondrial transcriptome. This has broadened the functional understanding of ncRNA in addition to their diversified modes of action in transcription regulation. The proposed model of 7S RNA in facilitating POLRMT dimerization is intriguing, but how does this ncRNA act during this process requires additional studies. This can be achieved by further polishing Cryo-EM resolution, i.e., with reduced 7S RNA conformational heterogeneity in the complex, to gain details in 7S RNA-POLRMT interactions. Finally, it is worthwhile noting that mitochondrial-linked diseases, such as Leigh syndrome and Leber hereditary optic neuropathy, are accompanied with transcriptional dysregulation [13], indicating that 7S RNA can be targeted as mitochondrial diagnostics and therapeutic purposes. Future studies will be of interest to profile how 7S RNA expression senses environmental or pathological changes to ultimately impact mtDNA transcription.
